# Implementation science in humanitarian assistance: applying a novel approach for humanitarian care optimization

**DOI:** 10.1186/s13012-024-01367-7

**Published:** 2024-05-29

**Authors:** Christopher W. Reynolds, Phillip J. Hsu, Dana Telem

**Affiliations:** 1 University of Michigan Medical School, 1301 Catherine St. Ann Arbor, Ann Arbor, MI 48109 USA; 2 Department of Surgery, University of Michigan, 1500 E Medical Center Dr. Ann Arbor, Ann Arbor, MI 48109 USA

**Keywords:** Humanitarian assistance, Global health, Implementation science, Quality improvement

## Abstract

Humanitarian assistance is hindered by a lack of strategies to optimize care delivery through research and organized networks. Distinct from global health, humanitarian assistance struggles to address its multifaceted challenges, including duplicative resources, uncoordinated communication, unregulated staff expertise and safety, financial waste, and poor-quality metrics and care delivery. Implementation science provides an exciting and underutilized approach that can be applied to address these challenges, by studying how to effectively design, implement, optimize, and scale evidence-based interventions. Though successful in well-resourced and global health systems, implementation science approaches are rare in humanitarian assistance. Adopting implementation science approaches including identifying determinants, creating accessible evidence-based intervention bundles, adapting study methodologies for the humanitarian context, and partnering with implementation experts could make these promising approaches more accessible for thousands of humanitarian actors delivering healthcare for millions of vulnerable patients worldwide.

**Supplementary Information:**

The online version contains supplementary material available at 10.1186/s13012-024-01367-7.

Contributions to the literature

- Humanitarian assistance is distinct from global health, with unique challenges that have gone largely unaddressed due to a lack of systematic approaches
- Implementation science is a successful approach to optimizing healthcare in well-resourced and global health settings but is much rarer in humanitarian assistance.
- By adapting implementation frameworks and methodologies to be more accessible for individuals working in humanitarian assistance, implementation science could be leveraged as one tool for designing approaches to delivering and scaling interventions to address healthcare challenges in the humanitarian field.

## Introduction

Humanitarian assistance, unlike global health, is hindered by limited pathways to optimize care through research and organized networks [1]. Global health is transnational study, research, and action to promote health equity [2]. Humanitarian assistance is more specific, constituting material and logistic assistance to vulnerable populations, including the homeless, refugees, and victims of war and famine. For more than 100 million displaced individuals and millions others in transitory states unable to access health systems, humanitarian assistance is critical [1]. However, a lack of concerted approaches in humanitarian assistance leads to wasted resources, unsafe practices, and perpetuation of mistakes, creating a blind spot to delivering quality care. Systematic approaches are needed to optimize research and care delivery in humanitarian settings.

Implementation science has been effective in global health and is a promising tool to design systems to overcome these challenges. Foundational components of implementation science including context dynamics, speed of response, and scaling can be key to effective humanitarian assistance delivery.

### Differentiation between humanitarian assistance and global health

While global health and humanitarian assistance share commonalities, their differences often go unrecognized. Global health prioritizes long-term partnerships to improve existing health systems, while humanitarianism operates outside of stable systems to deliver otherwise disrupted services [1]. Each differs in their approach, objectives, and temporality (Table 1). Challenges of unequal representation, task inefficiency, and wasted resources have affected both fields, which global health is addressing with accountability resources and movements towards decolonization [3]. Comparatively, humanitarian assistance has less reform, possibly attributable to limited professionalism opportunities and the conflation of humanitarianism with the humanitarian industrial complex [4]. Failing to recognize humanitarian assistance as distinct devalues the need for specific approaches to address its challenges.

**Table 1:** Definitions and differences between humanitarian assistance and global health

	**Humanitarian Assistance**	**Similarities**	**Global Health**
Environment	• Natural disaster, conflict and war zones, refugee camp settings	• “Acute on chronic” disasters^a^, disease outbreak (Ebola), conflict affected populations in LMICs	• Usually stable contexts in under-resourced health systems
Approach^b^	• Deliver absent services in chaotic settings, work in parallel to public or other organization health systems	• Low-resource settings, importance of professional development and ethics	• Integrated partnerships with existing health systems, oversite from Ministry of Health
Objectives	• Novel service delivery, limited stability in chaotic environments, bear witness to rights violations	• Improve health services for vulnerable populations	• Improve upon existing health services through training, access, research, and policy
Mechanisms	• Direct medical care, supporting and staffing clinics, evacuation, documentation of atrocities	• Training of local staff for acute needs, reciprocal learning, research collaborations	• Education, training and research capacity building, policy efforts, multi-institutional grants
Ownership	• Flexibility for international organizations to self-manage programs independently from in-country systems	• Space for co-design and mutual benefit	• Default to ownership by local stakeholders, operate with perspectives of being a “guest”
Temporality	• Most are short-term engagements, though time can range depending on setting and local context	• Protracted humanitarian disasters can extend long-term; educational or research global health initiatives can be finite	• Long-term partnership through actionable commitment should be the default engagement
Sustainability	• Service should last as long as it is needed in an acute disaster, not necessarily a need for longitudinal or sustained commitment	• Eventual transitions to local systems of operation	• Core tenant of equitable global health which should be integrated in nearly all efforts
Scope	• Intrinsic limitations of practice which should be acknowledged including resources and security	• Program effectiveness and reach often limited by funds or political agendas	• Less limitations for investment in longer-term initiatives and capacity building

Major differences and similarities between humanitarian assistance and global health

^a^ “Acute on chronic” is a term increasingly used to describe acute disasters which occur in areas with already damaged health systems. It is derived from the term in clinical medicine to describe an acute exacerbation of a chronic medical condition, but here used to describe what is observed when disasters affect already weakened country and regional health systems. Examples include the 2014–16 Ebola outbreak in West Africa, which was still struggling from the aftermath of decades of war and underfunded public health systems, Haitian earthquake in 2010, and the Turkish-Syrian earthquake further destabilizing displaced Syrian refugees

^b^Category definitions can be understood as the following: Environment describes the context in which global health or humanitarian assistance actions take place; approach describes the purpose and overarching method for engagement; Objectives describes actor goals in each setting; Mechanisms describes the specific interventions and programs employed in these settings; Ownership describes responsible actors within each context; Temporality highlights typical length of program involvement; Sustainability refers to necessity for longitudinal commitment within each context; and Scope describes the breadth or focus with which each field can be defined

### Implementation science in global health

Implementation science (IS) has been a promising approach to confront the challenges of global health. Aimed at reducing gaps from discovery to implementation through behavior change, IS focuses on five components: interventions, environments, behaviors, evaluation, and sustainability [5]. It enumerates not only which interventions are effective, but how and in which ways. These approaches have promoted innumerable evidence-based interventions in LMICs [5], and offer possibilities for cluster-randomized implementation trials, building from partnerships as seen through the ChEETAh trial [6]. IS models are also useful frameworks for conceptualizing historical issues plaguing global health, including ineffective implementation in diverse cultures, unsustainability, ignoring stakeholders, and insufficient scope [7].

Despite its impact within global health, IS remains foreign to humanitarianism. While a literature search of global health IS yielded thousands of references, the same for humanitarian assistance showed sparse results. Although global health institutes proliferate at universities and trainees are demanding opportunities, very few have humanitarian focuses or support researchers working in these settings. The etiologies behind this dearth of IS are multifactorial and likely include the urgency of crisis situations, reliance on resource-intense approaches not always available in such settings, and inaccessibility of IS expertise given its nascency, particularly outside of academic circles.

### Consequences from a lack of humanitarian assistance implementation science

Stark and plentiful examples define the challenges of humanitarian assistance. On February 6, 2023, a 7.8 magnitude earthquake struck Turkey and Syria, killing 50,000 people and displacing millions. Vast resources of human and financial capital were mobilized to address this disaster. As in most humanitarian contexts, it was easy to learn the number of dollars donated and actors responding: 102 countries offered assistance, 74 rescue teams were deployed, and two billion dollars were promised within two weeks [8]. Much harder to quantify is the impact of these resources. There is little data on the results, both positive and deleterious, these responses have had for affected persons in Turkey and Syria. Similar responses are seen with refugee crises affecting Western Hemisphere and Eastern European borders.

This begs the critical question: how effective are humanitarian systems? Objects of implementation in humanitarianism, including medical care; shelter; and water, sanitation, and hygiene (WASH), can be intuited as necessary for basic needs. Certainly, randomized-control trials are not always necessary for strong evidence, as it is clear how potable water reduces disease and timely surgical care prevents injurious complications [9]. However, there remains a startling dearth of evidence regarding effectiveness on objects of implementation in humanitarian assistance. Among few organizations that do evaluate these objects, efforts focus on singular interventions while overlooking integration into wider contexts. Systematic reviews on humanitarian objects of implementation are limited to one topic (i.e., maternal health) and conclude that rigorous methodologic approaches are rare [10]. While randomized-trials are not always ethical, creative approaches including natural quasi-experimental studies, interrupted time series, and difference-in-differences analyses could provide rigorous evaluation for implementation objects. One of the most comprehensive efforts to address these objects is the *Sphere Standards:* accepted humanitarian guidelines by which organizations can measure their effectiveness [11]. At minimum, organizations could benefit from evaluating their programs according to *Sphere*.

Without rigorous and iterative evaluation, advances in humanitarian assistance are stalled and mistakes repeat. Humanitarian workers lament limited coordination, insufficient preparation including language training, shortcomings in accomplishing objectives, duplicated services, violence towards workers, and wasted resources as perpetual failures. Authors Colombo and Pavignani attribute such failures to distant donor agendas, political and security obstacles, poor intercultural communication, and diverse epidemiological profiles [12]. Additionally, assistance organizations operate without universal measures of accountability or incentives for measuring true effectiveness. Premier organizations may track service metrics, including patient consultations, medications delivered, and funds going towards programming. However, such measures do not produce data that can be leveraged to improve health systems. Exemplar efforts by large, well-resourced organizations including operational research units within *Médecins sans frontières (*MSF) demonstrate successful system evaluation; yet these approaches remain the exception particularly for smaller organizations [13]. Patient health outcomes such as disability and quality-adjusted-life-years (DALYs/QALYs), cost-effectiveness, or mortality remain rare in humanitarian assistance [14].

### Implementation science approaches in humanitarian assistance

**Fig. 1:**
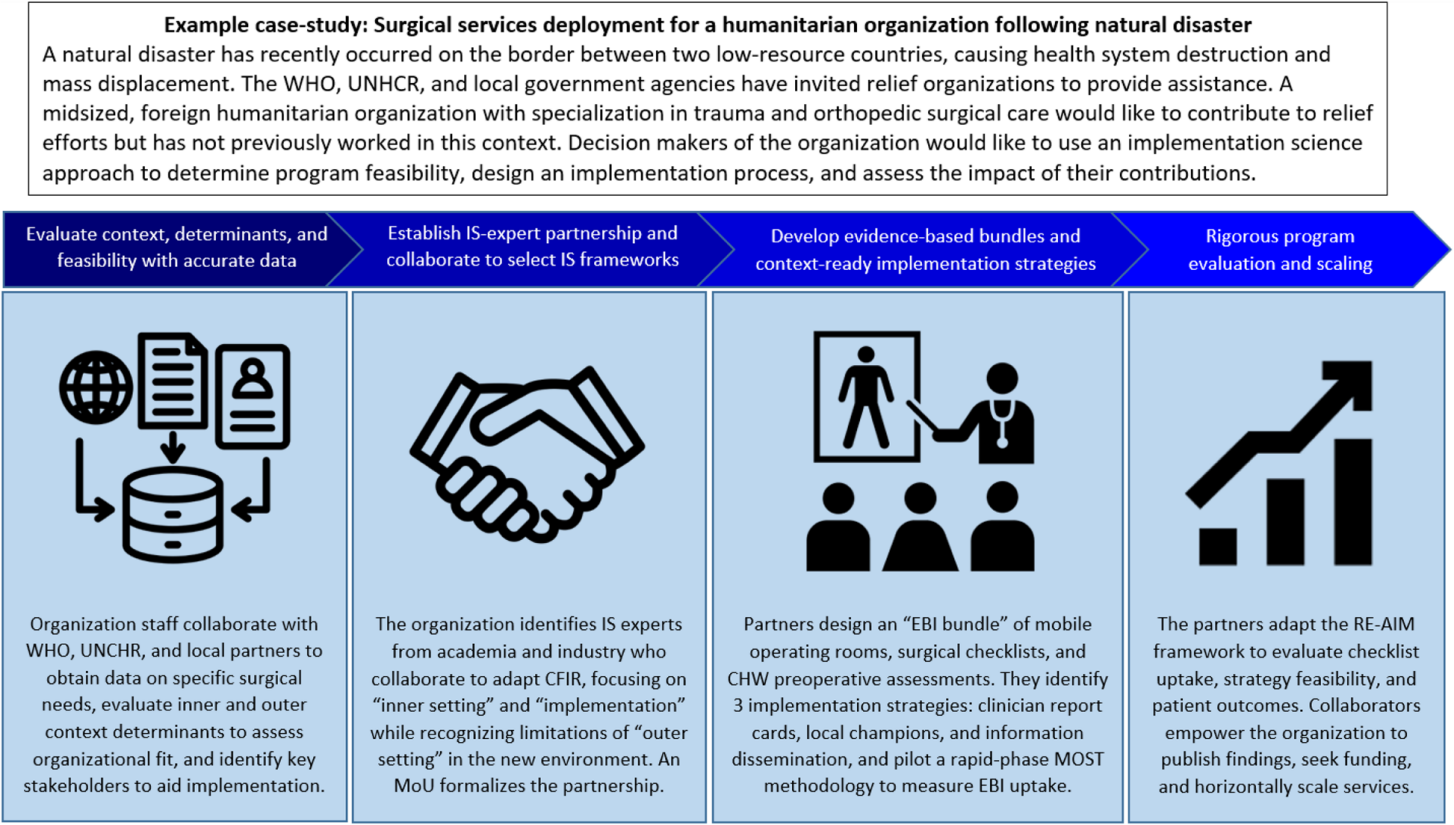
An implementation science approach to address a common situation in humanitarian assistance. This figure represents a process map demonstrating how an implementation science approach between a humanitarian assistance non-governmental organization and IS expert partners with research and pragmatic experience could contribute to solving a complex but common problem: rapid mobilization for surgical services following natural disaster. This example was created solely by the authors to demonstrate how IS could be leveraged in humanitarian settings and is not based on any specific organizations, clinicians, or patients. Abbreviations: CHW: community health worker, CFIR: Consolidated Framework for Implementation Research, EBI: evidence-based intervention, MOST: Multiphase Optimization Strategy Trial, MoU: Memorandum of Understanding, RE-AIM: Reach, Effectiveness, Adoption, Implementation, and Maintenance, UNHCR: United Nations High Commisioner for Refugees, WHO: World Health Organization

We propose implementation science as a solution to improve humanitarian assistance., The pitfalls of humanitarian assistance can be addressed through fundamentals of IS: problem identification, optimizing efficiency, iterative evaluation, conceptualizing context dynamics, adoption of evidence-based practices, speed of response, and scaling [4]. When made accessible, humanitarian assistance IS could optimize patient care and research to be feasible for organizations, while reflecting its integral components (Fig. 1).

#### Use of implementation science models for humanitarian context

Organizations can utilize IS theories, models, and frameworks for three aims: guiding processes for translating research into practice, explaining implementation outcomes, increasing coordination, and evaluating implementation [15]. Frameworks allow for conceptualization of inner and outer contexts to provide guidance on implementation feasibility, explain success or failures, and design or adapt evidence-based practices to local constraints [16]. Inner context describes attributes of organizations, while outer context defines the environment of operation. While experts agree that context is important, there is limited consensus on its definition [15]. We posit that, in humanitarian settings, context should be understood as a complex, dynamic system that is influenced not only by physical space and resources but also culture and power dynamics. Inner context for humanitarian organizations includes mission and goals, funding structures, temporal commitments, readiness for change, and communication networks. Outer contexts include the socio-political environment, safety and risks of operations, other services already provided, dynamics between other organizations, and external incentives. Both inner and outer contexts are highly important and demonstrate complex interplay. For example, an organization’s choice to engage in an outer context depends on internal resources, and once operating within a setting, that group’s characteristics can influence environment: a humanitarian group refusing to partner with other organizations may discourage collaboration among all actors.

Both inner and outer contexts influence implementation success and one another bidirectionally. However, outer contexts are often more difficult to navigate and likely have the strongest influence within humanitarian settings. Leveraging implementation models in humanitarian environments is a complicated task, and we believe that models which favor understanding outer context and system dynamics above intervention evidence are likely better suited to account for these variabilities. Models which emphasize flexibility and comprehensiveness through a few concise, core tenants are more likely feasible compared with rigid and complex ones. Example models which account for system dynamics while allowing flexibility include planned action models; Exploration, Preparation, Implementation, Sustainment (EPIS); and Implementation Climate, which can be supplemented by determinant frameworks as described by Nilsen [15]. Additionally, widely cited implementation and evaluation frameworks could be adapted for humanitarian use. While using a flexible model may be most accessible for humanitarian organizations, it may lack wide recognition in implementation literature. Adapting components of commonly referenced frameworks including The Consolidated Framework for Implementation Research (CFIR), Dynamic Sustainability Framework (DSF), and Promoting Action on Research Implementation in Health Services (PARIHS) to increase flexibility could benefit organizations to disseminate implementation successes through shared language [17] (Table 2, Supplementary material 1).

**Table 2:** Evaluation of common implementation models for their applicability and potential adaptations for use in humanitarian aid implementation science

Models	Pros	Cons	Applicability for humanitarianism^a^	Modifications for usability
Promoting Action on Research Implementation in Health Services (PARIHS)	• Moderate flexibility • Moderate scope (community, organizational, and system levels) • Context as centrally important • Three central components of evidence, context, and facilitation make interpretation accessible	• Incorporation of sub-elements risks overcomplication • Limited guidance for evaluation metrics	High	• Define targets for humanitarian implementation (populations, providers, governments) • Develop common outcome measurements depending on implementation objects
Exploration, Preparation, Implementation, Sustainment (EPIS)	• Assistance guides available • Synthesizes health problem of interest and existing evidence • Identifies determinants in preparation phase • Differentiates and accounts for inner and outer context • Acknowledges stakeholders including service environment • Explained in four manageable steps	• Less widely cited • Little guidance for outcome evaluation • Long-term sustainment not always appropriate	High	• Modify outer context dynamics with humanitarian specific factors (safety, community involvement, services from other organizations)
Implementation Climate	• Broad scope (individual, community, organizational, systems levels) • Recognizes context as important with inner and outer considerations • Highly effective for innovations that require collective behavior change	• Theory without sufficient implementation guidance • Intervention specific • Limited attention to evaluation outcomes	Medium/High	• Determine shared attributes for universalizing implementation approaches within multiple interventions • Assess if organizational culture is important for an aid organization’s workforce, especially if primarily short-term volunteers
Consolidated Framework for Implementation Research (CFIR)	• Widely referenced and understood in implementation science literatures • Many assistance tools to aid novel users • Context as highly important • Commonly used in LMICs • High construct availability to select for program specificity	• Narrow scope (community, organization levels) • Low flexibility • Many constructs increases complexity to master • Original CFIR lacks defined outcome measures	Medium/High	• Design or adapt constructs for humanitarian contexts, focused on community characteristics, safety, and external pressures including funding agent priorities and systems architecture
The Precede-Proceed Model	• Equal attention to dissemination and implementation • Moderate scope (community, organizational, individual) • Strong focus on evaluation metrics • Comprehensive prospective assessments (social, epidemiological, ecological) • Increases community ownership of programs	• Low flexibility • Complex and multifactorial • Multidimensional may be difficult for smaller organizations • Most evidence limited to educational interventions and chronic disease	Medium	• Limit most important aspects of each phase based on humanitarian goals
The RE-AIM Framework (Reach, Effectiveness, Adoption, Implementation, Maintenance/ Sustainment)	• Equal attention to dissemination and implementation • Strong focus on evaluation metrics • Moderate scope • Accessible language and evidence for real-world translation	• Low flexibility • Limited evidence synthesis • Context evaluation not a central component • Maintenance not always appropriate depending on context	Medium	• Supplement with proven evidence synthesis and a thorough evaluation of context determinants • Modify “Maintenance” according to goals and context

Evaluation of common IS frameworks for their applicability in humanitarian assistance

^a^Applicability for humanitarianism was determined by the authors’ evaluation of frameworks for their flexibility, comprehensiveness, resource intensity, diverse evaluation approaches, and frequency of its use in published studies in humanitarian assistance

Research to better understand inner and outer context dynamics could identify common determinants (barriers and facilitators) to implementation in humanitarian settings. While determinants will inevitably vary by organization, setting, resources, and scope, providing examples for actors to adapt known determinants and better understand their own is crucial to implementation [18].

#### Defining implementation, research, and scaling strategies for humanitarian contexts

Stakeholders could develop evidence-based intervention (EBI) bundles for use in humanitarian settings. The ERIC protocol defines 73 implementation strategies for EBI uptake, but few are feasible in resource-constrained environments [19]. Instead, a Delphi process to identify implementation strategy bundles in low-resource contexts could be beneficial. Bundles could empower organizations through common phases, including acute entry, collaboration with government and local partners, protection of health workers, transitions to local systems upon exit, fundraising, volunteer onboarding, and reporting metrics [1]. Short-term volunteers could benefit from behavior change EBI bundles which train to standards of care in local settings.

Timeliness is an additional factor limiting humanitarian research: once protocols are deployed following months-long development, pragmatic context could change. More creative methodologies could be validated, including adaptive randomized control trials, rapid-cycle multiphase optimization strategy trials (MOST), or Sequential Multiple Assignment Randomized Trials (SMART) with shorter randomization turnover to reduce study time [17]. For interventions with evidence, response speed and scaling are key components to consider in an IS-informed approach. Speed of response aligns with the fundamental objectives of humanitarian assistance, as shortening delivery time of life-saving services directly impacts outcomes. However, speed of response must be balanced with understanding context and implementation plans before involvement, as there are countless examples of failure due to uncoordinated responses. Improving coordination has been a key focus of recent humanitarianism. The World Health Organization regularly organizes “health clusters” within disasters, and supervisory offices including the United Nations High Commissioner for Refugees and government departments now assign roles to organizations before arrival. On meso- and macro-levels, IS tenants could increase coordination and decrease response time. One method is to identify diverse stakeholders with comprehensive frameworks such as the 7P’s (providers, patients/public, payers, purchasers, product developers, policymakers, principal investigators) to engage interdisciplinary groups beyond usual responders. Overlapping fields including quality improvement and management sciences can also work within an overarching IS framework to improve coordination, especially as new actors become involved. Organizations could provide synopses of inner context including strategic plans, specialty areas, and resource capacity before initiating a response, while coordinating supervisors pilot horizontal dissemination strategies to communicate key messages.

The process of scaling validated interventions in humanitarian settings could also utilize IS approaches. Scaling in fragile areas depends heavily on context and dynamics, including resources and commitment of organizations (inner), temporality and scope of the disaster (outer), and political priorities of coordinating bodies (mixed). While vertical scaling is often a goal in global health and non-fragile settings, this approach is not always best in humanitarian contexts. More applicable is horizontal scaling due to the typical absence of necessary services across entire spectrums. Through horizontal scaling, organizations with effective supply and care systems could enhance services by optimizing existing mechanisms through expanded scope. Horizontal scaling could also combat vertical evaluation of singular programs and shift to assess impact within a complete system.

#### Pathways to actualize implementation science in humanitarian contexts

Infrastructure should be built to track accurate, applicable, and accountable metrics. Process and delivery outcomes can be effective for measuring implementation success and should continue to be valued. For organizations with capacity, developing infrastructure to track clinical outcomes can improve the standard by which actors evaluate their impact. While not an innovation of IS, rigorous data collection and evaluation is a routine piece to most frameworks, and those commonly used including RE-AIM and Precede-Proceed should be employed by organizations with feasible data management approaches [15]. Innovative technologies, including geospatial and satellite mapping, machine learning for epidemic models, mHealth, and electronic health records can be integrated into existing systems [20]. Evaluation metrics feasible for smaller organizations, including chart reviews and qualitative analysis, should be incentivized with publication and grant opportunities. By adopting an implementation approach specific to resource-poor settings, organizations could study available resources as “primary research objects,” rather than “resources as context” [16]. Similarly, the implementation of structures can be viewed as an intervention for evaluation since success depends equally on structures as objects themselves [9]. Motivating organizations to highlight implementation structures can be similarly beneficial for advancing reproducible knowledge.

These recommendations can occur with mutually beneficial humanitarian organization—IS expert partnerships [14]. Such experts are found in diverse fields including academia, business, and NGOs. Smaller organizations lack infrastructure to independently establish evaluation projects, but even well-known, highly-resourced organizations could benefit from collaboration, as shown in global health [7]. Academic partners could co-design implementation frameworks for use by humanitarian organizations, who receive data to optimize health delivery. Analytical and publication support would raise organizations’ profiles for larger grants, while industry partnerships offer innovation and sustainable funding mechanisms. NGO partners could benefit from shared lessons to improve their own services, while academics publish valuable data while centering careers around vulnerable patients. Health students yearning for opportunities to serve humanitarian populations could do so as trainees. Existing mechanisms could formalize these partnerships, including Memorandums of Understanding, data sharing agreements, and bidirectional exchange for lectures and professional development.

While IS shows exciting promise for humanitarian assistance, its use could bring potential disadvantages. The introduction of new approaches and actors could unintentionally exacerbate poor coordination if done without intentionality for organization. The feasibility of complex research designs requires adaptation for crisis situations, and interdisciplinary partnership across multiple institutions, particularly when partnering with academia, could slow the scale-up of services. Being informed about both positives and complications that IS can have on assistance delivery will allow actors to mitigate potential disadvantages and make informed decisions on whether these approaches are appropriate for context.

## Conclusion

Humanitarian assistance is a complex field with a crucial aim: care for the world’s most vulnerable populations. Addressing its longstanding deficiencies will require organized approaches and recognition as a distinct discipline. Implementation science is a promising solution to optimize care and research for these vulnerable populations but necessitates substantial adaptation and partnership for feasibility in humanitarian settings. The potential successes make this task worth pursuing, most importantly for the millions of patients who receive healthcare from humanitarian organizations.

### Supplementary Information

Supplementary Material 1.

## Data Availability

Not applicable.

## Abbreviations

CFIR: Consolidated Framework for Implementation Research,
ChEETAh: Routine sterile glove and instrument change at the time of abdominal wound closure to prevent surgical site infection
DALYs: Disability-adjusted-life-years
DSF: Dynamic Sustainability Framework
EBI: Evidence-based intervention
EPIS: Exploration, Preparation, Implementation, Sustainment
ERIC: Expert recommendations for implementing change
HIV: Human immunodeficiency virus
LMIC: Low and middle-income countries
MOST: Multiphase Optimization Strategy Trial
PARIHS: Promoting Action on Research Implementation in Health Services
QALYs: Quality-adjusted-life-years
RE-AIM: Reach, Effectiveness, Adoption, Implementation, and Maintenance
SMART: Sequential Multiple Assignment Randomized Trials
TICD: Tailored Implementation in Chronic Disease
