# Supplementary material for: Implementation science in humanitarian assistance: applying a novel approach for humanitarian care optimization
Source: Implement Sci. 2024 May 29;19:38. doi: 10.1186/s13012-024-01367-7 (PMC11138019; doi:10.1186/s13012-024-01367-7)
Supplement: Supplementary file 1 — Supplementary Material 1. [file 13012_2024_1367_MOESM1_ESM.docx]

Table 2: Evaluation of common implementation models for their applicability and potential adaptations for use in humanitarian aid implementation science

| Models | Pros | Cons | Applicability for humanitarianism^a^ | Modifications for usability |
| --- | --- | --- | --- | --- |
| Promoting Action on Research Implementation in Health Services (PARIHS)^b^ | - Moderate flexibility - Moderate scope (community, organizational, and system levels) - Context as centrally important - Three central components of evidence, context, and facilitation make interpretation accessible | - Incorporation of sub-elements risks overcomplication - Limited guidance for evaluation metrics | High | - Define targets for humanitarian implementation (populations, providers, governments) - Develop common outcome measurements depending on implementation objects |
| Exploration, Preparation, Implementation, Sustainment (EPIS) | - Assistance guides available - Synthesizes health problem of interest and existing evidence - Identifies determinants in preparation phase - Differentiates and accounts for inner and outer context - Acknowledges stakeholders including service environment - Explained in four manageable steps | - Less widely cited - Little guidance for outcome evaluation - Long-term sustainment not always appropriate | High | - Modify outer context dynamics with humanitarian specific factors (safety, community involvement, services from other organizations) |
| Implementation Climate | - Broad scope (individual, community, organizational, systems levels) - Recognizes context as important with inner and outer considerations - Highly effective for innovations that require collective behavior change | - Theory without sufficient implementation guidance - Intervention specific - Limited attention to evaluation outcomes | Medium/High | - Determine shared attributes for universalizing implementation approaches within multiple interventions - Assess if organizational culture is important for an aid organization’s workforce, especially if primarily short-term volunteers |
| Consolidated Framework for Implementation Research (CFIR) | - Widely referenced and understood in implementation science literatures - Many assistance tools to aid novel users - Context as highly important - Commonly used in LMICs - High construct availability to select for program specificity | - Narrow scope (community, organization levels) - Low flexibility - Many constructs increases complexity to master - Original CFIR lacks defined outcome measures | Medium/High | - Design or adapt constructs for humanitarian contexts, focused on community characteristics, safety, and external pressures including funding agent priorities and systems architecture |
| The Precede-Proceed Model | - Equal attention to dissemination and implementation - Moderate scope (community, organizational, individual) - Strong focus on evaluation metrics - Comprehensive prospective assessments (social, epidemiological, ecological) - Increases community ownership of programs | - Low flexibility - Complex and multifactorial - Multidimensional may be difficult for smaller organizations - Most evidence limited to educational interventions and chronic disease | Medium | - Limit most important aspects of each phase based on humanitarian goals |
| The RE-AIM Framework (Reach, Effectiveness, Adoption, Implementation, Maintenance/ Sustainment) | - Equal attention to dissemination and implementation - Strong focus on evaluation metrics - Moderate scope - Accessible language and evidence for real-world translation | - Low flexibility - Limited evidence synthesis - Context evaluation not a central component - Maintenance not always appropriate depending on context | Medium | - Supplement with proven evidence synthesis and a thorough evaluation of context determinants - Modify “Maintenance” according to goals and context |
| Institutional Change Theory | - Determines organizational readiness for change - Recognizes complexity for organizational implementation at individual and system levels - High flexibility | - Theory without sufficient implementation guidance - May be more useful for large, highly-structured organizations | Medium | - Supplement with guides to conceptualize outer dynamics - Pair with determinant identification process |
| Pronovost’s 4E’s Process Theory | - Comprehensive in preparation and outcome evaluation - Easy to understand, four-step process | - Designed for high-resource system implementation - Limited evidence in LMICs - Focused heavily on individual behavior change within a system - Limited guidance on how to design toolkits and structure iterative evaluation systems | Medium | - Integrate intervention toolkit design within preparation phases - Include community stakeholders in design beyond determinant identification |
| Interactive Systems Framework (ISF) | - Equal attention to dissemination and implementation - Moderate flexibility - Broad scope (system, community, organization, and individual levels) - Step-by-step process to advance through each prevention system | - Complex interplay between prevention synthesis/translation, support, and delivery systems - Favors comprehensiveness above targeted implementation - Context not well accounted for | Low/Medium | - Incorporate context analysis as fourth system - Add government and safety level factors as implementation influences to already defined individual, organizational, and community factors - Protocolize when to advance through prevention systems so organizations understand each’s role |
| Practical, Robust Implementation and Sustainability Model (PRISM) | - Context highly important - Highly comprehensive | - Narrow scope - Low flexibility - Highly complex approach risks overcomplicating implementation - Not all contextual factors integral to PRISM are knowable (sustainability infrastructure, external environment) | Low/Medium | - Pairing the contextual analysis from PRISM with implementation and evaluation outcome of RE-AIM would be highly useful |
| Dynamic Sustainability Framework (DSF) | - Context and the changing nature of environments as highly influential factors in implementation - Moderate flexibility - Ongoing adaptation of interventions overtime | - Heavy focus of sustainability which is not always necessary in humanitarian aid - Attention on vertical scaling - Limited recommendations for manipulating the ecological system if interventions are incompatible | Low/Medium | - Helpful for organizations planning to pilot and adapt desired interventions overtime - Provide nuanced approaches for planned length of sustainability |
| Normalization Process Theory | - Moderate flexibility - Broad scope (system, community, organization, individual levels) - Incorporation of context - Accounts for determinants - Studies how to incorporate routine interventions to the point of normalization | - Focus mainly on individual behavior and inner context - Individual motivations may be less impactful areas for organizations relying on short-term volunteers - Designed as a tool for trialists which may be less useful for humanitarian actors - Lack of linearity for evaluating dynamic relationships may overcomplicate implementation analysis | Low/Medium | - Define first which interventions in humanitarian context would benefit from normalization - Modify NPT to account for environmental components including feasibility, population reach, and strategic operations |
| Situated Change Theory | - Environment as highly important - Recognizes multifactorial influences on context - Historical precedence as impact on system dynamics - Reduces risk of implementing inappropriate or duplicated services | - Underestimates organizational agency in influencing context - Risks slow care delivery when speed is valued | Low | - Pair with an implementation model to enact change while understanding theory behind its uptake |
| Tailored Implementation in Chronic Disease (TICD) | - Widely cited - Strong focus on determinants - Tangible recommendations for interventions to address determinants of practice | - Created specifically for chronic disease management in primary care settings - Highly laborious process to reproduce completely or adapt - Evidence limited on effect of primary and secondary outcomes - Insufficient account of context | Low | - Adapt intervention recommendations to humanitarian context, populations, and problems - Study modifications and effects across different contexts and disease states |
| A Six-Step Framework for International Physical Activity Dissemination | - Moderate flexibility - Broad scope (system, community, organization, individual, policy levels) - High focus on preparation including evidence synthesis and stakeholder engagement | - Dissemination focused without implementation specifics - Not widely referenced | Low | - Consider use by agencies which advise organizations, to disseminate findings from practical implementation - Use specifically for educational and health promotion interventions or combine with a more focused implementation approach |

Table 2 footnote: Evaluation of common IS frameworks for their applicability in humanitarian assistance. ^a^Applicability for humanitarianism was determined by the authors’ evaluation of frameworks for their flexibility, comprehensiveness, resource intensity, diverse evaluation approaches, and frequency of its use in published studies in humanitarian assistance.

^b^References for each of the included frameworks are listed:

PARIHS: Rycroft-Malone J. The PARIHS framework--a framework for guiding the implementation of evidence-based practice. J Nurs Care Qual. 2004 Oct-Dec;19(4):297-304. doi: 10.1097/00001786-200410000-00002.

EPIS: Moullin JC, Dickson KS, Stadnick NA, Rabin B, Aarons GA. Systematic review of the Exploration, Preparation, Implementation, Sustainment (EPIS) framework. Implement Sci. 2019 Jan 5;14(1):1. doi: 10.1186/s13012-018-0842-6.

Implementation climate: Powell BJ, Mettert KD, Dorsey CN, Weiner BJ, Stanick CF, Lengnick-Hall R, Ehrhart MG, Aarons GA, Barwick MA, Damschroder LJ, Lewis CC. Measures of organizational culture, organizational climate, and implementation climate in behavioral health: A systematic review. Implement Res Pract. 2021 Jun 21;2:26334895211018862. doi: 10.1177/26334895211018862.

CFIR: Damschroder LJ, Aron DC, Keith RE, Kirsh SR, Alexander JA, Lowery JC. Fostering implementation of health services research findings into practice: a consolidated framework for advancing implementation science. Implement Sci. 2009 Aug 7;4:50. doi: 10.1186/1748-5908-4-50.

Precede-Proceed: Crosby R, Noar SM. What is a planning model? An introduction to PRECEDE-PROCEED. J Public Health Dent. 2011 Winter;71 Suppl 1:S7-15. doi: 10.1111/j.1752-7325.2011.00235.x.

RE-AIM: Glasgow RE, Vogt TM, Boles SM. Evaluating the public health impact of health promotion interventions: the RE-AIM framework. Am J Public Health. 1999 Sep;89(9):1322-7. doi: 10.2105/ajph.89.9.1322.

Institutional Change Theory: Joyce ML, Cronin JJ Jr. An application of institutional change theories in the healthcare industry: implications for planning. J Health Care Mark. 1985 Spring;5(2):9-18.

4E’s Process Theory: Pronovost PJ, Berenholtz SM, Needham DM. Translating evidence into practice: a model for large scale knowledge translation. BMJ. 2008 Oct 6;337:a1714. doi: 10.1136/bmj.a1714.

ISF: Wandersman A, Duffy J, Flaspohler P, Noonan R, Lubell K, Stillman L, Blachman M, Dunville R, Saul J. Bridging the gap between prevention research and practice: the interactive systems framework for dissemination and implementation. Am J Community Psychol. 2008 Jun;41(3-4):171-81. doi: 10.1007/s10464-008-9174-z.

PRISM: Feldstein AC, Glasgow RE. A practical, robust implementation and sustainability model (PRISM) for integrating research findings into practice. Jt Comm J Qual Patient Saf. 2008 Apr;34(4):228-43. doi: 10.1016/s1553-7250(08)34030-6.

DSF: Chambers DA, Glasgow RE, Stange KC. The dynamic sustainability framework: addressing the paradox of sustainment amid ongoing change. Implementation Sci. 2013;8,117. doi.org/10.1186/1748-5908-8-117

Normalization Process Theory: Murray E, Treweek S, Pope C, MacFarlane A, Ballini L, Dowrick C, Finch T, Kennedy A, Mair F, O'Donnell C, Ong BN, Rapley T, Rogers A, May C. Normalisation process theory: a framework for developing, evaluating and implementing complex interventions. BMC Med. 2010 Oct 20;8:63. doi: 10.1186/1741-7015-8-63.

Situated Change Theory: Dadich A, Doloswala N. What can organisational theory offer knowledge translation in healthcare? A thematic and lexical analysis. BMC Health Serv Res. 2018 May 10;18(1):351. doi: 10.1186/s12913-018-3121-y.

TICD: Wensing M. The Tailored Implementation in Chronic Diseases (TICD) project: introduction and main findings. Implement Sci. 2017 Jan 10;12(1):5. doi: 10.1186/s13012-016-0536-x.

Six-Step Framework for International Physical Activity Dissemination: Bauman AE, Nelson DE, Pratt M, Matsudo V, Schoeppe S. Dissemination of physical activity evidence, programs, policies, and surveillance in the international public health arena. Am J Prev Med. 2006 Oct;31(4 Suppl):S57-65. doi: 10.1016/j.amepre.2006.06.026.
